# Optimization Study on the Two-Color Injection Molding Process of Medical Protective Goggles Based on the BP-SSA Algorithm

**DOI:** 10.3390/polym18050613

**Published:** 2026-02-28

**Authors:** Ming Yang, Yasheng Li, Jubao Liu, Feng Li, Jianfeng Yao, Sailong Yan

**Affiliations:** 1 Mechanical Science and Engineering College, Northeast Petroleum University, Daqing 163318, China; yangm@nepu.edu.cn (M.Y.); 13952351947@163.com (Y.L.); qaz1774632@163.com (S.Y.); 2 Hongfeng Intelligent Manufacturing (Shenzhen) Co., Ltd., Shenzhen 518117, China; lifeng_hangking@163.com (F.L.); yjf_hangking@163.com (J.Y.)

**Keywords:** medical goggles, two-color injection molding, process optimization, moldflow simulation, BP-SSA model, warpage deformation

## Abstract

To solve common defects such as warpage deformation, interface debonding, and uneven filling during the two-color injection molding of medical goggles while meeting their multi-performance requirements, including high light transmittance, impact resistance, chemical corrosion resistance, and structural stability, this study conducts research on the process optimization of two-color injection molding. Firstly, based on the principle of material compatibility and Moldflow simulation, a suitable material combination was selected: the first-shot frame adopts Apec 1745 PC material, and the second-shot lens uses Makrolon 2858 PC material, which effectively avoids the risk of interface non-fusion. Subsequently, a high-precision 3D simulation model was established using Moldflow software, and the injection sequence of “frame first, lens second” was optimized and determined. A gating system with double-gate (for the frame) and single-gate side feeding (for the lens), as well as a cooling system with an 8 mm diameter, was designed, and all key indicators of mesh quality meet the simulation requirements. Taking the mold and melt temperatures, holding pressures, and holding times of the two shots as design variables and warpage deformation as the optimization objective, sample data were obtained through an *L*_32_ (7^4^) orthogonal test. A BP neural network was constructed to describe the nonlinear relationship between parameters and quality, and the Sparrow Search Algorithm (SSA) was combined to optimize the weights and thresholds of the network, forming a BP-SSA intelligent optimization model. The results show that the mean absolute percentage error (MAPE) of the proposed model is only 2.28%, which is significantly better than that of the single BP neural network (14.36%). The optimal process parameters obtained by optimization are a mold temperature of 130 °C, first-shot melt temperature of 311 °C, second-shot melt temperature of 310 °C, first-shot holding pressure of 83 MPa, second-shot holding pressure of 70 MPa, first-shot holding time of 14 s, and second-shot holding time of 8 s. Simulation and mold test verification indicate that after optimization, the warpage deformation of the goggles is reduced to 0.8956 mm (simulation) and 0.944 mm (measured), with a relative error of only 5.4%, which is 67.9% lower than the initial simulation result. The integrated method of “material selection—CAE simulation—orthogonal test—BP-SSA intelligent optimization” proposed in this study provides technical support for the high-precision manufacturing of thin-walled transparent multi-material medical products.

## 1. Introduction

In scenarios such as medical diagnosis, treatment, and nursing care, medical goggles serve as core protective equipment to safeguard healthcare workers’ eyes from splashes, aerosols, and contaminants, and their performance is directly linked to the occupational safety of users [1]. With the continuous improvement of medical protection standards, modern medical goggles are required to meet multiple stringent criteria simultaneously: splash and leakage resistance to block pathogen transmission, lightweight comfort to meet long-term wearing needs, durability to maintain protective efficacy after repeated disinfection, and the use of materials that are free from harmful substances and comply with medical biocompatibility standards [2]. However, traditional manufacturing processes struggle to balance these performance requirements: single-color injection molding cannot simultaneously achieve high impact resistance for the frame and high light transmittance for the lens; assembled or bonded structures suffer from poor sealing performance and are prone to interface aging. Following repeated disinfection, such structures are likely to experience debonding and leakage, which seriously impairs protective reliability.

To overcome these limitations, two-color injection molding technology, as an advanced multi-material molding process, enables the sequential injection of two plastics with distinct properties into a single mold via a dedicated two-color injection molding machine, realizing one-step forming. This technology achieves seamless integration of products, thus featuring strong structural integrity, high production efficiency, and excellent design flexibility, which perfectly meet the core performance requirements of medical goggles [3]. However, during the two-color injection molding process, factors including material compatibility, injection sequence, and process parameter matching tend to induce defects such as interface debonding, uneven filling, and warpage deformation [4]. Particularly for the 1.5 mm thick, thin-walled transparent structures of medical goggles, the uneven distribution of temperature fields and stress fields will exacerbate warpage deformation, seriously affecting the optical performance and structural accuracy of the product. In addition, interrelated aspects, including material compatibility selection, gating system, and cooling system design, and process parameter matching further increase the difficulty of process optimization.

To address the aforementioned challenges in two-color injection molding, extensive research has been conducted on process optimization. Shi et al. [5] carried out a two-color molding simulation of the two-color front shell of liquid crystal televisions using Moldflow software 2019. Using the orthogonal experimental method, they obtained an optimized set of process parameters with the sink mark index and volumetric shrinkage rate during ejection as response indicators. Miao et al. [6] targeted a specific two-color plastic buckle, reducing the warpage deformation from 0.5583 mm to 0.2733 mm by increasing the local wall thickness of the product, adding more gates, and implementing a decaying holding pressure strategy. However, most of these studies depend on traditional statistical optimization methods or a single simulation tool, making it challenging to accurately depict the highly nonlinear relationships between multiple process parameters and molding quality.

To address this issue, the backpropagation (BP) neural network, due to its robust nonlinear approximation capability, has been extensively utilized to establish mapping models between injection molding process parameters and quality characteristics. Hong et al. [7] proposed a multi-objective optimization approach based on the BP neural network and NSGA-II algorithm for junction box housings, obtaining the optimal combination of process parameters. As a result, the volumetric shrinkage rate and warpage deformation were reduced by 33.2% and 3.8%, respectively. Wang et al. [8] established a back propagation artificial neural network prediction model for the gate freeze time of injection-molded polypropylenes, which fully verified the reliable nonlinear fitting capability of BP neural networks in predicting key process parameters of injection molding. Through Moldflow simulation and experimental verification, BP-based hybrid algorithms have been proven to effectively improve the molding accuracy of thin-walled injection-molded parts, with warpage and volumetric shrinkage reduced significantly. Lin et al. [9] further combined the backpropagation neural network with particle swarm optimization to conduct multi-objective process parameter optimization, identifying the optimal parameters and improving product quality. Zhang et al. [10] proposed a multi-objective optimization method for injection molding energy consumption and product quality using a combined approach of response surface methodology (RSM) and BP neural network, verifying the strong applicability of BP neural network combined with RSM in solving multi-objective optimization problems involving injection molding process parameters (energy consumption reduction and product quality improvement). Li et al. [11] realized warpage optimization of fiber-reinforced composite injection molding by combining the backpropagation neural network and a genetic algorithm, expanding the application of intelligent algorithms to composite materials. Liu et al. [12] conducted multi-objective optimization of injection molding process parameters for moderately thick plane lenses based on PSO-BPNN, OMOPSO, and TOPSIS, providing a complete multi-objective decision framework. Zhang et al. [13] achieved multi-objective optimization of injection molding process parameters based on Opt LHD, EBFNN, and MOPSO, enriching the technical path of intelligent optimization. Song et al. [14] took an automotive wiring harness protection frame as the research object, developed a two-hidden-layer BP neural network (BPNN) model based on orthogonal experimental design and response surface methodology (RSM), and optimized the weights and thresholds of the BPNN model using the genetic algorithm (GA). This approach effectively reduced the warpage and volumetric shrinkage of thin-walled components. Yin et al. [15] integrated the BP neural network with the GA to develop a multi-objective optimization model, and case analysis showed that the proposed method could adjust process parameters accurately and effectively to meet the requirements of actual production. Peng et al. [16] developed a DBO-BP neural network prediction model for thin-walled connectors based on simulation results and response data, which enhanced the accuracy of multi-objective optimization, optimized injection molding process parameters, improved production efficiency, and reduced defects. Li et al. [17] proposed a parallel integrated learning technique based on improved particle swarm optimization (PSO) and a BP neural network, which effectively enhances the optimization efficiency and prediction accuracy of complex systems. When applied to process parameter optimization, this technique achieves precise control of product quality indicators, further verifying the application potential of improved PSO-BP hybrid algorithms in injection molding. Wang et al. [18] optimized and calibrated the model parameters of the back-propagation (BP) neural network through the ant colony optimization (ACO) algorithm, established a precise mathematical model between the injection molding process parameters and the molding quality objectives, subsequently completed the multi-objective global optimization of the model by combining the non-dominated sorting genetic algorithm (NSGA-II), and finally obtained the optimal combination of process parameters, which effectively solved the quality control problems in the injection molding of fiber-reinforced composites.

Nonetheless, the BP neural network algorithm tends to trap into local optima, which restricts its prediction accuracy [19]. Although algorithms such as PSO, ACO and GA have been employed to enhance the BP neural network [8,18,20], the Sparrow Search Algorithm (SSA), a novel swarm intelligence optimization algorithm, offers advantages including robust global search capability and rapid convergence speed [21]. Integrating the SSA with the nonlinear fitting capability of the BP neural network can effectively enhance the prediction and optimization accuracy of complex systems. Recent studies have verified the feasibility of BP-SSA hybrid algorithms. Zhao et al. [22] established the nonlinear mapping relationship between four-dimensional decision variables and three-dimensional key output variables via the SSA-BP neural network model, carried out multi-objective optimization using the NSGA-II algorithm, and determined the optimal operating condition from the Pareto frontier by the entropy weight-TOPSIS method. Ling et al. [23] established a multi-layer BP neural network to model the drying kinetics and energy consumption characteristics, and optimized the network weights and threshold parameters using the SSA. Comparative analysis demonstrated that the SSA-optimized BP neural network was significantly superior to the traditional BP model and the genetic algorithm-optimized model in prediction accuracy. Liu et al. [24] proposed a novel parameter calibration framework for the storm water management model (SWMM) model based on the SSA and BPNN.This framework used SSA to provide the optimal initial weights and thresholds for BPNN, constructing an efficient and stable SSA-BPNN surrogate model.This surrogate model can characterize the mapping relationship between the uncertain parameters of the SWMM model and the node water depth, thereby rapidly optimizing the parameter combination of the SWMM model and improving the model fitting accuracy. Zhang et al. [25] addressed the shortcomings of traditional BP neural networks (i.e., insufficient prediction accuracy caused by random initial weights and thresholds and proneness to local optima) by introducing the SSA to optimize the initial network parameters and constructing the SSA-BP model, which effectively improved the convergence speed and generalization ability of the model. The results demonstrated that compared with the traditional BP model, the prediction error of the SSA-BP model was reduced by 53.4%; the model possessed superior prediction accuracy and could effectively characterize the nonlinear relationships affected by multiple factors. Li et al. [26] combined SSA and BP neural network to construct a prediction model for the tensile strength of welded joints, and established a multi-objective optimization model based on Non-dominated Sorting Genetic Algorithm II (NSGA-II) and Technique for Order Preference by Similarity to Ideal Solution (TOPSIS). Using the TOPSIS-based multi-objective optimization method, the optimal parameter combinations were determined with ultimate tensile strength and first debonding strength as the optimization objectives. However, its application in two-color injection molding of thin-walled transparent medical products remains insufficiently explored.

To address these gaps, this study focuses on medical goggles as the research subject. First, suitable material combinations for two-color injection molding are selected through material compatibility simulation. Subsequently, a high-precision Moldflow 3D CAE simulation model is developed to optimize the gating system, cooling system, and injection sequence. Sample data relating to process parameters and warpage deformation are acquired through orthogonal experiments, and a BP-SSA intelligent optimization model is established to realize global optimization of the process parameters. Finally, the engineering feasibility of the optimized process is validated through mold trials. This study aims to resolve the core technical challenges associated with the two-color injection molding of medical goggles, provide technical support for the high-quality production of thin-walled, transparent, and multi-material medical devices, and broaden the application scope of intelligent optimization algorithms in the field of advanced manufacturing.

## 2. Materials and Methods

### 2.1. Experimental Objects and Material Properties

#### 2.1.1. Structural Parameters of Medical Goggles

**Figure 1:**
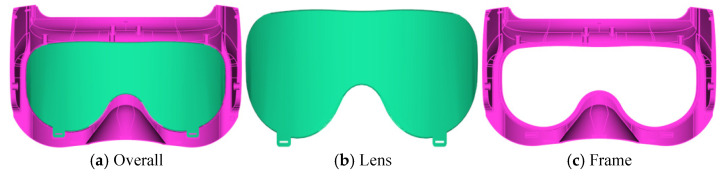
3D model of the medical goggles.

In this study, medical goggles were selected as the research object, and their three-dimensional (3D) model was constructed using UG software, as illustrated in Figure 1. The goggles are primarily composed of two components: the frame and the lens. The maximum overall dimensions are 143.47 mm × 82.26 mm × 70.88 mm, with a uniform wall thickness of 1.5 mm across the entire structure. Specifically, the lens has dimensions of 122.82 mm × 55.1 mm × 34.56 mm and a volume of 9.34 cm^3^, which must satisfy the requirements of high light transmittance and surface finish (free from defects such as scratches and bubbles). The frame has a volume of 23.14 cm^3^ and is required to possess excellent impact resistance and structural stability to ensure the secure fixation of the lens. Owing to the presence of numerous holes and cylindrical features in the goggle frame, the issue of uneven injection molding filling is likely to occur. This defect needs to be mitigated through simulation and process optimization.

#### 2.1.2. Material Selection for Two-Color Injection Molding

**Figure 2:**
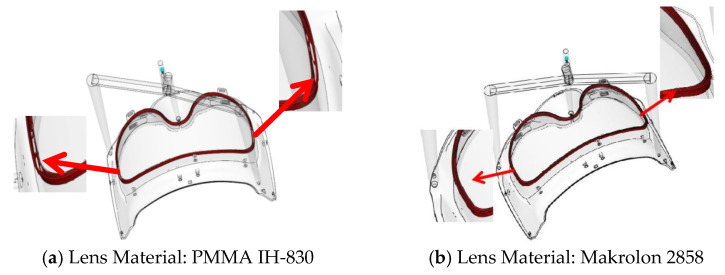
Remelted interface at the overmolding joint.

**Figure 3:**
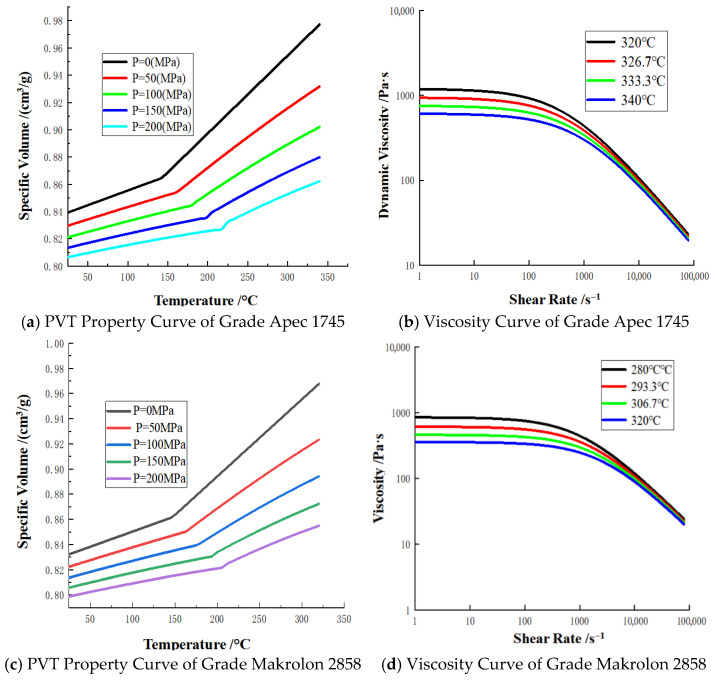
Material property curves.

Based on the performance requirements of medical goggles and the material compatibility principles of two-color injection molding, the injection materials were screened. Considering that medical goggles undergo frequent cleaning and disinfection during service, they are required to exhibit excellent durability to ensure that their protective performance remains uncompromised after multiple disinfection cycles. Polycarbonate (PC) resin of grade Apec 1745 was selected as the first-shot material for the frame, which possesses ultra-high impact resistance and chemical corrosion resistance. The second-shot material for the lens must satisfy three critical criteria: high transparency, good compatibility with the first-shot material, and a melt temperature lower than that of the first-shot material. The candidate materials included PC resin of grade Makrolon 2858 and polymethyl methacrylate (PMMA) of grade IH-830. Through overmolding simulation via Moldflow software, as depicted in Figure 2, it was observed that unmelted regions existed at the interface between PMMA IH-830 and Apec 1745, which are prone to inducing adhesion failure defects. In contrast, no interfacial unmelding phenomenon was detected between Makrolon 2858 and Apec 1745. The Pressure–Volume–Temperature (PVT) property curves and viscosity property curves of Apec 1745 and Makrolon 2858 are presented in Figure 3. At the same temperature, the two materials exhibit comparable specific volume and viscosity, with Makrolon 2858 having a slightly lower viscosity than Apec 1745. Their favorable compatibility facilitates the formation of an integrated structure, reduces delamination and interfacial tension, and thereby mitigates the risk of warpage deformation.

### 2.2. Establishment of CAE Simulation Model for Two-Color Injection Molding

#### 2.2.1. Meshing

**Figure 4:**
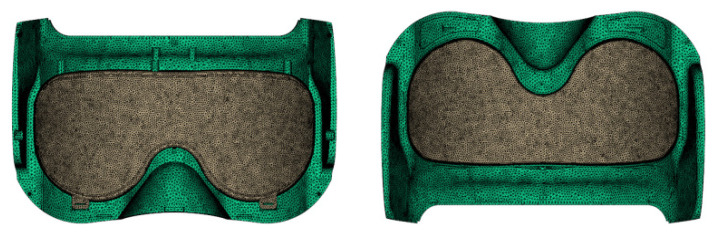
Mesh model.

Simulation was performed using Moldflow 2019 software. Given that the warpage deformation analysis of two-color injection molding requires the complete capture of the temperature and stress field distributions within the 3D cavity, the 3D mesh type was selected. However, to repair model defects, the dual-domain mesh was first chosen after model import, and the exact matching option was selected in the assembly model contact surface settings to ensure mesh consistency at the contact interfaces of the two-color product. After eliminating all model defects, the mesh type was converted to a 3D mesh, followed by re-meshing. With a uniform model thickness of 1.5 mm, the mesh edge length is typically set to 1–2 times the thickness of the injection-molded part. To accurately capture detailed features such as holes and cylinders, a numerical model was established with a global mesh edge length equal to 1 times the wall thickness. The total number of meshes was 843,330, with a minimum number of layers of 6. For 3D meshes, inverted tetrahedral elements and overlapping faces must be absent; additionally, the maximum edge length ratio should be less than 3, and the maximum-to-average-volume ratio must be less than 20. The quality of the 3D meshing is presented in Table 1, which indicates that the mesh quality meets the simulation requirements. The mesh model is illustrated in Figure 4.

#### 2.2.2. Determination of Injection Sequence

If the transparent lens is used as the first shot, the high-temperature melt of the frame (second shot) will cause the lens to remelt. This disrupts the frozen state of macromolecular chain orientation, resulting in local stress mutations and ultimately inducing warpage deformation and internal stress imbalance. In contrast, using the frame as the first shot avoids thermal damage to the frame due to the lower melt temperature of the lens (second shot), and the flow residual stress is more likely to distribute uniformly. Therefore, the injection sequence was determined as follows: the frame is injected first, followed by the lens.

#### 2.2.3. Gating System Design

##### Selection of Gate Location and Quantity

**Figure 5:**
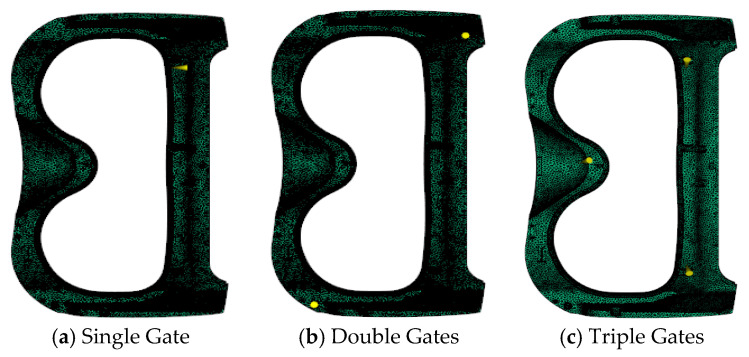
Gate locations. Colored dots indicate the gate locations.

**Figure 6:**
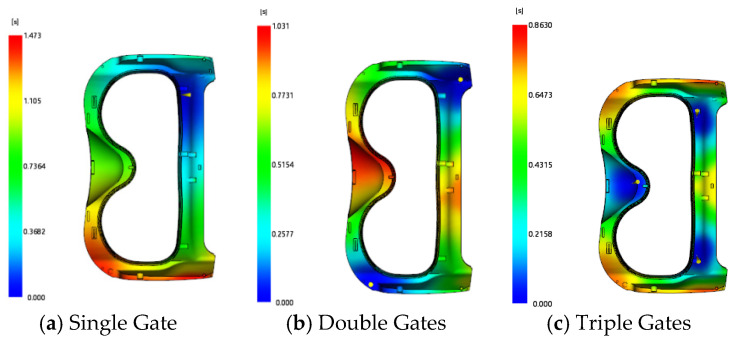
Filling time cloud diagrams.

**Figure 7:**
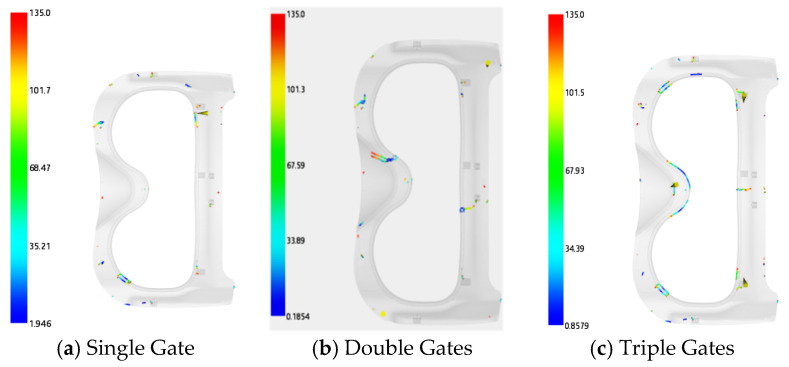
Frame weld line cloud diagrams.

**Figure 8:**
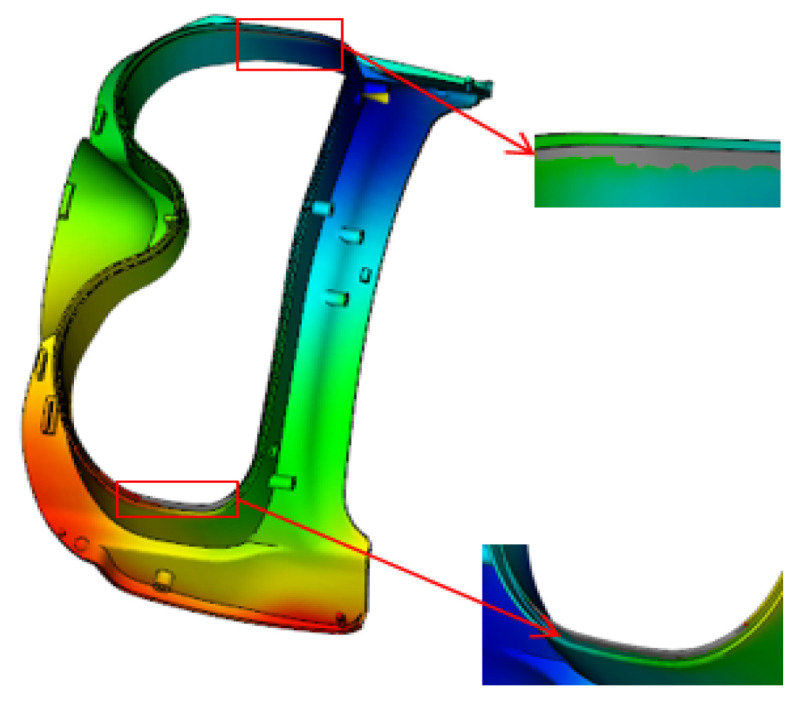
Single-gate short shot cloud diagram.

For the frame injection-molded part, three gating schemes were proposed: a single gate, double gates, and triple gates. The gate locations are illustrated in Figure 5. The filling cloud diagrams of the three schemes are shown in Figure 6: the filling time is 1.472 s for the single-gate scheme, 1.031 s for the double-gate scheme, and 0.863 s for the triple-gate scheme. The weld line cloud diagrams are presented in Figure 7, indicating that the single-gate scheme has the fewest weld lines, while the triple-gate scheme has the most. However, a short shot defect was observed during the filling process of the single-gate scheme (see Figure 8). Thus, the double-gate feeding method was selected for the frame injection-molded part.

**Figure 9:**
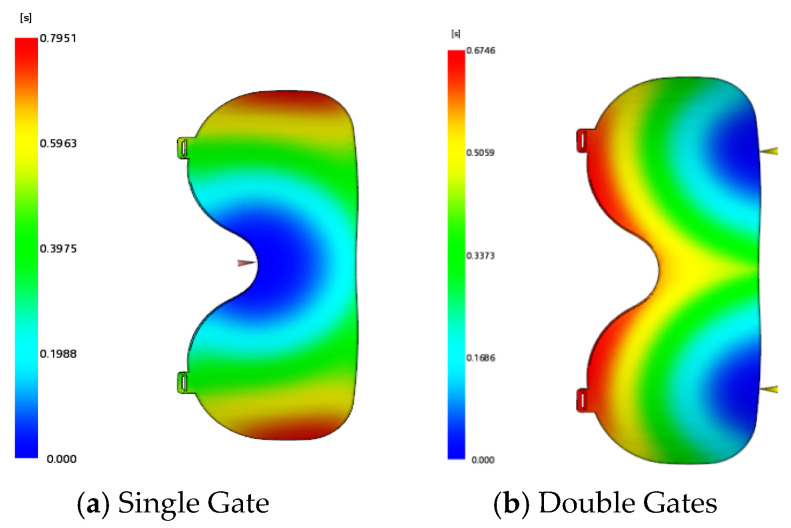
Gate locations and filling time result cloud diagrams.

**Figure 10:**
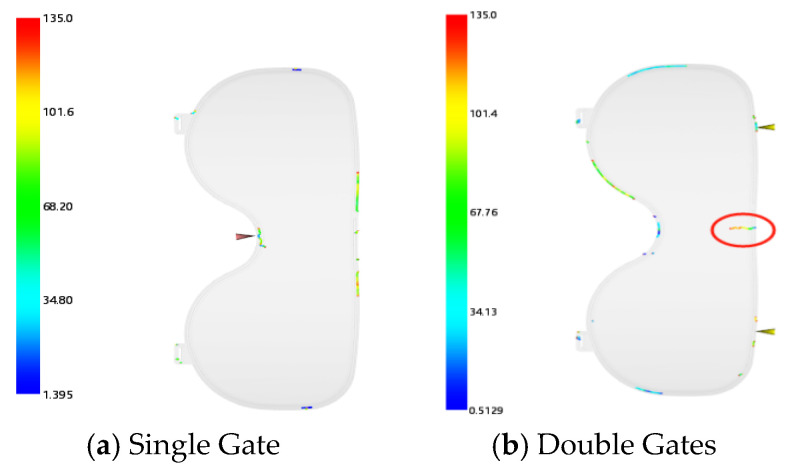
Lens weld line cloud diagrams.

Considering the transparency of the lens, surface feeding is prone to causing flow marks; thus, side feeding was adopted. Two gating schemes were established: single gate and double gate. The gate locations and filling time result cloud diagrams are illustrated in Figure 9. The filling time for the double-gate scheme is 0.6746 s, while that for the single-gate scheme is 0.7951 s. The weld line cloud diagrams of the two schemes are presented in Figure 10. The double-gate scheme generates weld lines on the surface layer of the lens, whereas the weld lines of the single-gate scheme are distributed around the lens and covered by the frame. Therefore, the single-gate side-feeding method was selected for the lens injection-molded part.

##### Runner Size Calculation

The injection-molded part is a small, thin-walled component, adopting a one-cavity mold design. Therefore, a straight sprue was selected, and there are two independent gating systems (one for each shot). The calculation formula for the sprue diameter is shown in Equation (1):

(1) $$D={\left(\frac{8}{\pi \gamma }\right)}^{\frac{1}{3}}*{\left(\frac{3n+1}{n}*\frac{V}{tN}\right)}^{\frac{1}{3}}$$

where *D* is the sprue diameter, *γ* is the shear rate, *n* is the non-Newtonian index, *V* is the injection volume, *t* is the filling time, and *N* is the number of runner branches.

For the first-shot (frame) gating system, which is equipped with two gates, the number of runner branches is 2. The volume of the frame is 23.14 cm^3^, and the filling time is 1.031 s. The non-Newtonian index of the frame material was obtained from material data sheets as 0.288, and the sprue shear rate was set to 1200 s^−1^. After calculation and rounding up, the sprue diameter for the first shot was determined to be 6 mm.

For the second-shot (lens) gating system, featuring one gate, the number of runner branches is 1. The volume of the lens is 9.34 cm^3^, with a filling time of 0.7951 s. The non-Newtonian index of the lens material was retrieved from material data sheets as 0.17, and the sprue shear rate was also specified as 1200 s^−1^. Through calculation and rounding up, the sprue diameter for the second shot was similarly determined to be 6 mm.

The calculation formula for the runner diameter is shown in Equation (2):

(2) $$d_{j+1}=\frac{d_{j}}{N_{j+1}^{\frac{1}{3}}}$$

where *d_j_* is the sprue diameter, *d_j_*_+1_ is the runner diameter, and *N_j_*_+1_ is the number of lower-layer runner branches.

The sprue diameter of the frame is 6 mm, with 2 runner branches. The frame runner diameter was determined to be 5 mm by calculation and rounding up. For the lens, the sprue diameter is 6 mm, and the number of runner branches is 1; thus, the lens runner diameter was determined to be 6 mm through calculation. A trapezoidal runner geometry was adopted, as it can reduce the velocity gradient and pressure loss.

#### 2.2.4. Cooling System Design

The cooling system was designed based on heat transfer theory, with water as the cooling medium. The calculation formula for the heat released by the molten plastic is shown in Equation (3):

(3) $$Q_{\mathrm{in}}=nmh$$

where *Q*_in_ is the heat transferred to the mold, *n* is the number of injections per hour of the injection molding machine, *m* is the injection mass per shot, and *h* is the specific enthalpy of the plastic.

The injection molding machine is expected to perform 43 injections per hour with a cycle time of 82 s. The total injection mass per shot is 53.12 g, including 40.5 g for the frame and 12.62 g for the lens. The specific enthalpy of the material was obtained from material data sheets as 1250 kJ/kg. Through calculation using the formula, the heat released by the molten plastic was determined to be 2855.2 kJ/h.

Assuming that the heat carried away by the coolant is equal to the heat released by the plastic, the volume flow rate q was calculated using Equation (4):

(4) $$Q_{in}=q\rho c\Delta T$$

where *ρ* is the density of water (1000 kg/m^3^), *c* is the specific heat capacity of water (4.18 kJ/(kg·°C)), and Δ*T* is the temperature difference between the inlet and outlet of the coolant (5 °C).

Through calculation, the volume flow rate was determined to be 2.3 × 10^−3^ m^3^/min. Referring to the relationship between coolant flow velocity and pipe diameter, when *q* < 5 × 10^−3^ m^3^/min, the pipe diameter is selected as 8 mm, with a pipe spacing of 20 mm and a distance of 16 mm from the cavity wall.

### 2.3. Process Parameter Optimization Method

#### 2.3.1. Orthogonal Experiment Design

The two-color goggles are thin-walled transparent parts, and the product structures of the first and second shots differ significantly. The selection of process parameters has a significant impact on the product molding quality. Unlike the molding process of single-color products, a two-color injection molding machine performs injection molding independently through different nozzles of the same mold. Therefore, the mold temperature remains consistent during the mold clamping and opening process, while different parameter selections are required for the melt temperature, holding pressure, and holding time of the first and second shots. Finally, the mold temperature (A), first-shot melt temperature (B), second-shot melt temperature (C), first-shot holding pressure (D), second-shot holding pressure (E), first-shot holding time (F), and second-shot holding time (G) were determined as the design variables, and part warpage (K) was selected as the output variable. The value ranges of mold temperature and melt temperature were set in accordance with material recommendations.

**Figure 11:**
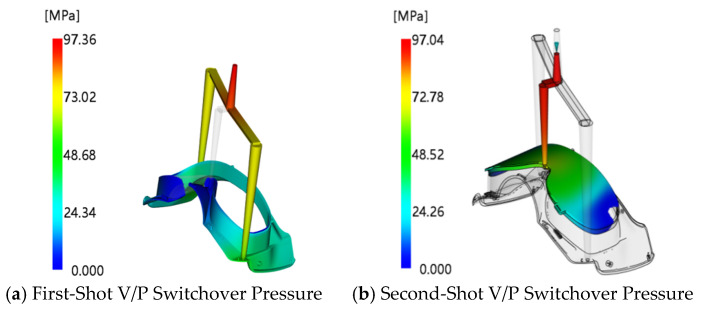
V/P switchover pressures of the injection-molded part.

Through simulation analysis, the V/P switchover pressure for the first shot was found to be 97.36 MPa, and that for the second shot was 97.04 MPa, as shown in Figure 11. The holding pressure is generally selected as 70–100% of the V/P switchover pressure; thus, the holding pressure range for both the first and second shots is 68–97 MPa. The holding time should not exceed the gate freezing time minus the part filling time. Therefore, the first-shot holding time should not exceed 16.72 s (rounded up to 17 s), and the second-shot holding time should not exceed 10 s. Taking warpage deformation as the evaluation index, the factors and levels of the designed orthogonal experiment are shown in Table 2. The *L*_32_ (7^4^) orthogonal array was adopted, which requires only 32 experiments.

#### 2.3.2. BP Neural Network Construction

The BP neural network possesses powerful nonlinear function approximation capability, enabling it to accurately describe the relationship between injection molding part quality and injection process parameters [27]. It consists of an input layer, a hidden layer, and an output layer. Each node in the previous layer is fully connected to all nodes in the subsequent layer, with no interconnections between nodes in the same layer. The calculation process includes both forward and backward propagation. The transfer function is shown in Equation (5). The number of input layer nodes is set to 7, corresponding to the 7 design variables, and the number of output layer nodes is set to 1, which refers to the warpage deformation (K).

(5) $$Sigmiod(x)=\frac{1}{1+e^{-x}}$$

**Figure 12:**
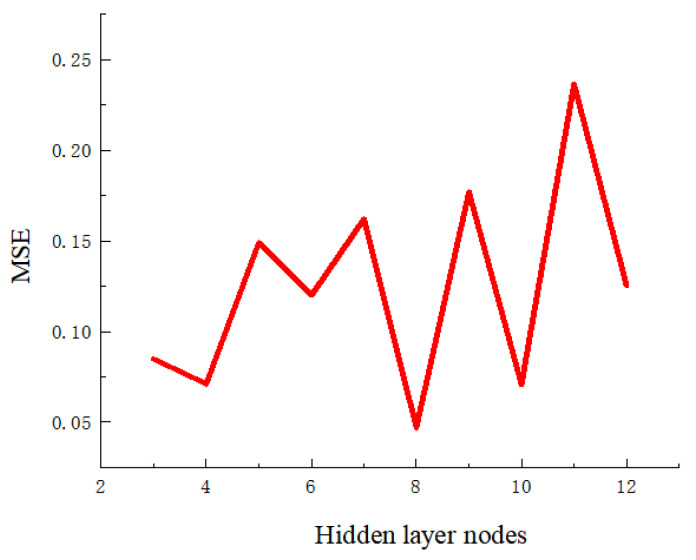
Relationship curve between the number of hidden layer nodes and MSE.

When designing the BP neural network, it is necessary to carefully balance the size of the hidden layer to ensure the network has both sufficient expressive capability and computational efficiency. In this paper, the selection of the hidden layer is calculated using Equation (6):

(6) $$m=\sqrt{n+t}+\alpha$$

where *m* is the number of hidden layer neurons, *n* is the number of input layer neurons, *t* is the number of output layer neurons, and α is a constant within [1, 10]. The relationship curve between the number of hidden layer neurons and the mean squared error (MSE) established in this paper is shown in Figure 12. It can be seen from the figure that the MSE reaches the minimum value when the number of hidden layer neurons is 8. Thus, the number of hidden layer neurons in the proposed neural network is determined to be 8.

After the neural network parameters and sample selection were completed, the BP neural network model was established using MATLAB software, and the parameter settings of the neural network model are shown in Table 3.

#### 2.3.3. Global Optimization of Process Parameters Using the Sparrow Search Algorithm

The Sparrow Search Algorithm (SSA) is a novel swarm optimization algorithm proposed by Xue [28]. Inspired by the foraging and anti-predation behaviors of sparrows, it is a new type of swarm intelligence optimization algorithm. The initial positions of the sparrow population can be expressed in the form of a matrix, as shown in Equation (7):

(7) $$X=\left[\begin{array}{cccc} x_{1,1} & x_{1,2} & \ldots & x_{1,\dim}\\ x_{2,1} & x_{2,2} & \ldots & x_{2,\dim}\\ \vdots & \vdots & \ddots & \vdots \\ x_{n,1} & x_{n,2} & \ldots & x_{n,\dim} \end{array}\right]$$

Its fitness value can be expressed using Equation (8):

(8) $$f_{x}=\left[\begin{array}{cccc} f[x_{1,1} & x_{1,2} & \ldots & x_{1,\dim}]\\ f[x_{2,1} & x_{2,2} & \ldots & x_{2,\dim}]\\ \vdots & \vdots & \ddots & \vdots \\ f[x_{n,1} & x_{n,2} & \ldots & x_{n,\dim}] \end{array}\right]$$

In a population, each member is assigned a fitness value that reflects their ability to solve the problem. In this context, discoverers refer to individuals with higher fitness values during foraging. In the process of solving the problem, each individual in the population updates their position in the problem space by comparing their fitness values with each other. This position update process aims to find the optimal solution to the problem, or at least a solution close to the optimal one. In this way, individuals with higher fitness values have a greater chance of being retained and participating in the next round of iteration, ultimately achieving the optimization goal of problem-solving. The position update formula for discoverers is shown in Equation (9):

(9) $${x^{t+1}}_{i,j}=\left\{\begin{array}{cc} x_{i,j}^{t}\cdot \exp\left(\frac{-i}{\alpha \cdot T_{\max}}\right), & R_{2}<ST\\ x_{i,j}^{t}+Q\cdot L, & R_{2}\geq ST \end{array}\right.$$

where *t* is the number of iterations, and *x^t^_i,j_* denotes the *j*-th dimensional position of the *i*-th individual in the *t*-th generation of the sparrow population. When *R*_2_ < ST, it indicates that foraging is safe and large-scale foraging can be conducted, while when *R*_2_ ≥ ST, it means that scouts detect danger and issue warnings, prompting discoverers to move to safe areas, and followers will move following the positions of discoverers to obtain better food resources, with the position update formula for followers in the sparrow population shown in Equation (10):

(10) $${x^{t+1}}_{i,j}=\left\{\begin{array}{cc} Q\cdot \exp(\frac{x_{\mathrm{worst}}^{t}-x_{i,j}^{t}}{i^{2}}), & i>n/2\\ x_{p}^{t+1}+\left|x_{i,j}^{t}-x_{p}^{t+1}\right|\cdot A^{+}\cdot L, & \mathrm{otherwise} \end{array}\right.$$

where *x^t^*_worst_ denotes the worst fitness position in the *t*-th generation of the population, *x^t^*^+1^*_i_*_,*j*_ denotes the best fitness position of the sparrow in the *t* + 1-th generation of the population, *A*^+^ is a 1× dim matrix, with *A*^+^ = *A^T^*(*AA^T^*)^−1^, and *n* is the population size. When *i* > *n*/2, it indicates that the fitness of the individual is relatively low, and it needs to forage elsewhere; otherwise, the follower will forage around the optimal individual.

In the sparrow population, a portion of individuals exhibit vigilance behaviors while foraging to detect potential dangers. When they perceive possible threats, they will abandon their current foraging behavior and switch to searching for the next foraging site. The proportion of individuals engaging in such vigilance behaviors in the entire population is fixed, typically ranging from 10% to 20%, to ensure the overall safety of the population. The position update method for vigilantes in the sparrow population is shown in Equation (11):

(11) $$x_{i,j}=\left\{\begin{array}{cc} {x^{t}}_{best}+\beta \cdot \left|{x^{t}}_{i,j}-{x^{t}}_{best}\right|, & f_{i}>f_{g}\\ {x^{t}}_{i,j}+K\cdot \left(\frac{\left|{x^{t}}_{i,j}-{x^{t}}_{worst}\right|}{(f_{i}-f_{w})+\varepsilon }\right), & f_{i}=f_{g} \end{array}\right.$$

**Figure 13:**
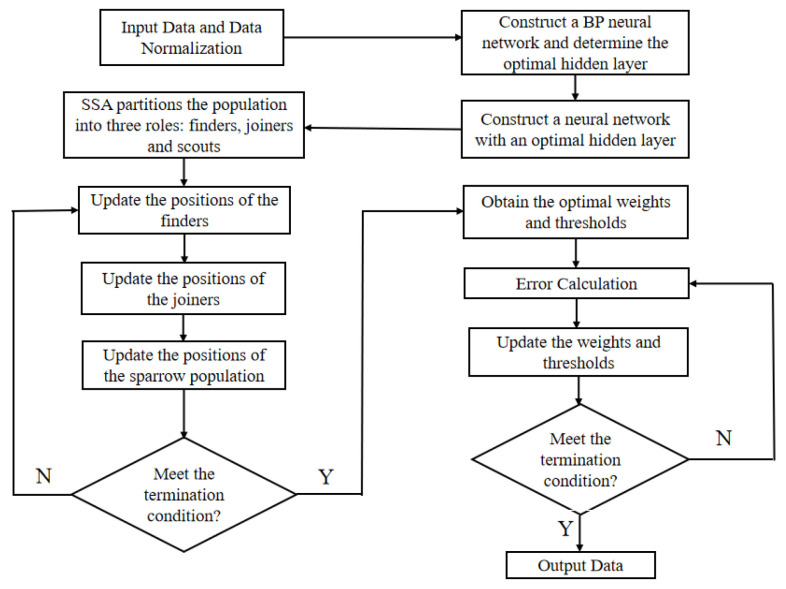
Global optimization flowchart of the SSA-optimized neural network.

The established BP neural network is optimized using the SSA, with the neural network model serving as the fitness function and the minimum warpage deformation as the optimization objective of the SSA to search for optimal process parameters. The optimization flow chart is shown in Figure 13.

### 2.4. Injection Molding Machine Parameters

**Figure 14:**
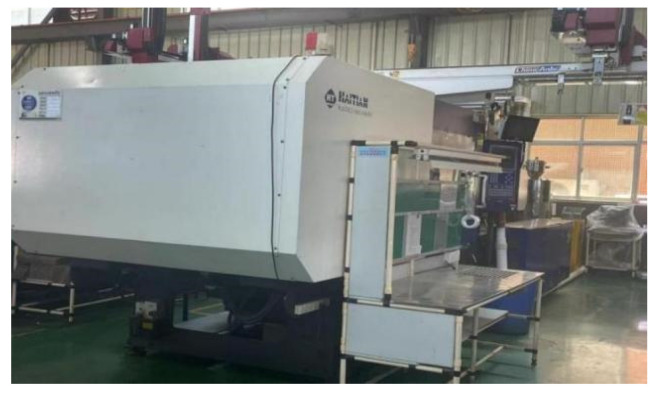
Haitian two-color injection molding machine.

Two different grades of materials were respectively injected into the barrels of the injection molding machine for production mold trial verification. The injection molding machine used in production is a two-color injection molding machine from the Haitian Group, with the IA-2500 model and a clamping force of 250 tons (T). The injection molding machine is shown in Figure 14, and its relevant parameters are listed in Table 4.

## 3. Results and Discussion

### 3.1. Analysis of Initial Simulation Results

**Figure 15:**
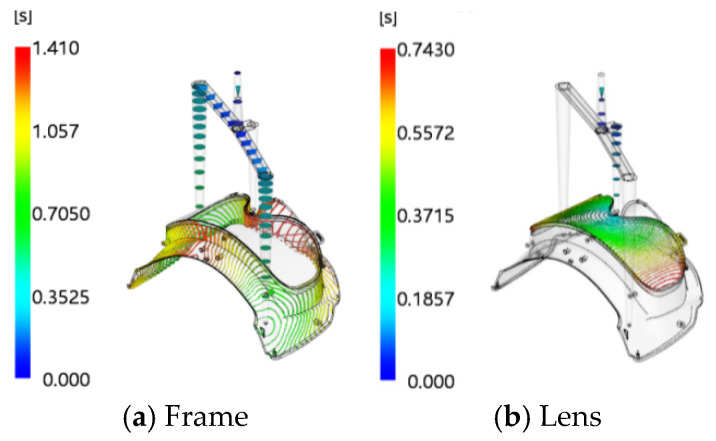
Contour clouds of filling time for the injection-molded part.

**Figure 16:**
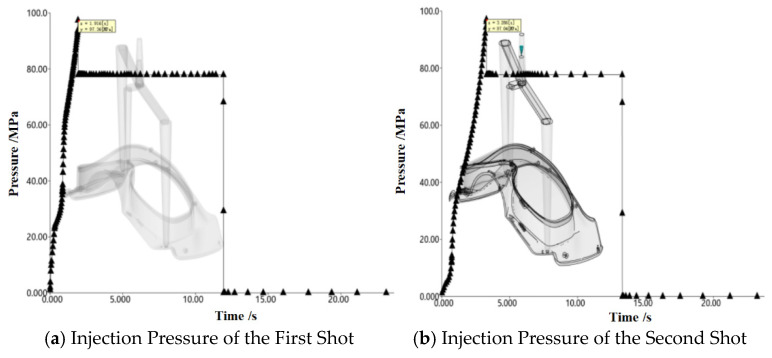
Pressure–time curves at the injection positions.

**Figure 17:**
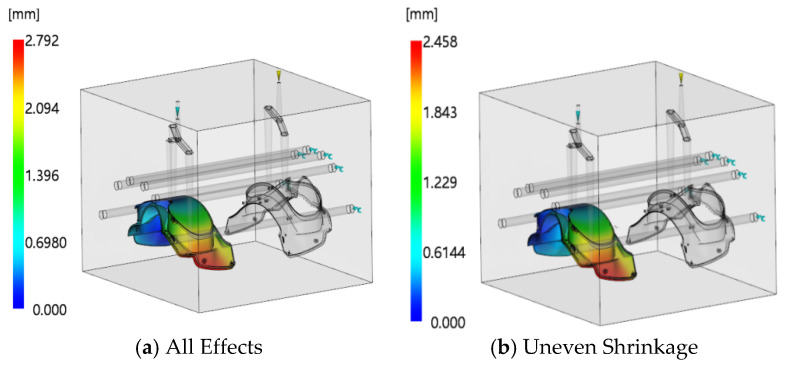
Contour clouds of warpage deformation for the injection-molded part.

Initial simulations were performed based on the established numerical model. The mold temperature and melt temperature of the injection-molded part were set to the material-recommended temperatures provided by Moldflow software, and the holding pressure was initially set to 80% of the V/P switchover pressure. The contour clouds of filling time for the first- and second-shot-molding parts are shown in Figure 15. It can be seen from the figure that the filling time of the first shot is 1.41 s and that of the second shot is 0.743 s. The uniform distribution of contour lines indicates that the velocity distribution of the melt at the flow front is balanced. At the end of filling, all parts of the cavity are almost fully filled simultaneously at the distal end, reflecting a smooth filling process of the melt in the cavity with an overall well-balanced flow state. The pressure–time curves at the injection positions are presented in Figure 16. As indicated in the figure, the maximum pressure of the first shot is 97.36 MPa, and that of the second shot is 97.04 MPa, both of which are lower than the maximum injection pressure of the injection molding machine. At the initial stage of injection molding, the injection pressure reaches its peak; as time progresses, the pressure curve gradually flattens out, indicating that the melt flow in the cavity has stabilized. Under this state, the melt will not overflow the cavity boundary due to excessive pressure, which effectively reduces the risk of molding defects such as flash and provides a reliable guarantee for the molding of high-quality two-color injection-molded products. The warpage analysis results are shown in Figure 17. The maximum warpage deformation is 2.792 mm, among which the deformation caused by uneven shrinkage is 2.458 mm. This does not meet the production requirements and thus requires further optimization.

### 3.2. Orthogonal Test Results

Taking the overall warpage deformation of the injection-molded part as the optimization objective, simulations were conducted on the injection-molded part according to the data in the test table (as shown in Table 5), and the simulation results are presented in the table. The warpage deformation values show significant differences under different combinations of process parameters, ranging from 0.963 mm to 3.46 mm. Among the parameters, mold temperature and the holding pressure of the first shot have the most significant impact on warpage deformation. An increase in mold temperature can reduce material shrinkage differences, while reasonable matching of the holding pressure of the first shot can minimize residual internal stress.

### 3.3. Optimization Results of the BP-SSA Model

Twenty-seven groups of orthogonal test data were used as the training set, with five groups used as the test set. The mean absolute percentage error (MAPE) of the BP neural network prediction was 14.36%, while after optimization with the SSA, the MAPE dropped to 2.28%, indicating a significant improvement in prediction accuracy, as shown in Table 6. This is because the SSA avoids the BP network falling into local optima through a global search strategy and enhances the model’s fitting ability for nonlinear relationships. It also verifies the accuracy of the established BP-SSA optimization model.

**Figure 18:**
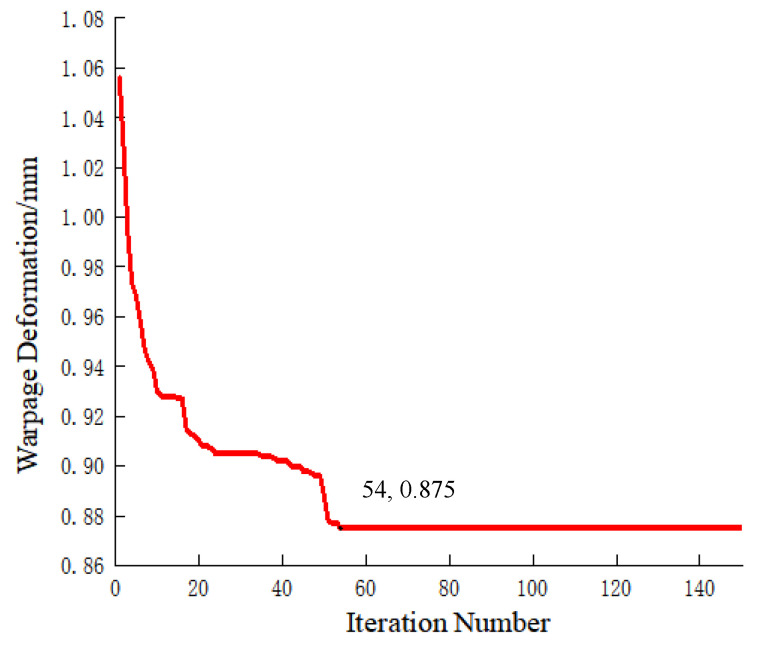
Iteration curve.

Global optimization of process parameters was performed using the BP-SSA model, with the iteration curve shown in Figure 18. The objective value reached its minimum at the 54th iteration, and the optimal process parameters are as follows: mold temperature of 130 °C, first-shot melt temperature of 311 °C, second-shot melt temperature of 310 °C, first-shot holding pressure of 83 MPa, second-shot holding pressure of 70 MPa, first-shot holding time of 14 s, and second-shot holding time of 8 s, with the corresponding predicted warpage deformation value of 0.875 mm.

### 3.4. Simulation and Mold Trial Verification

**Figure 19:**
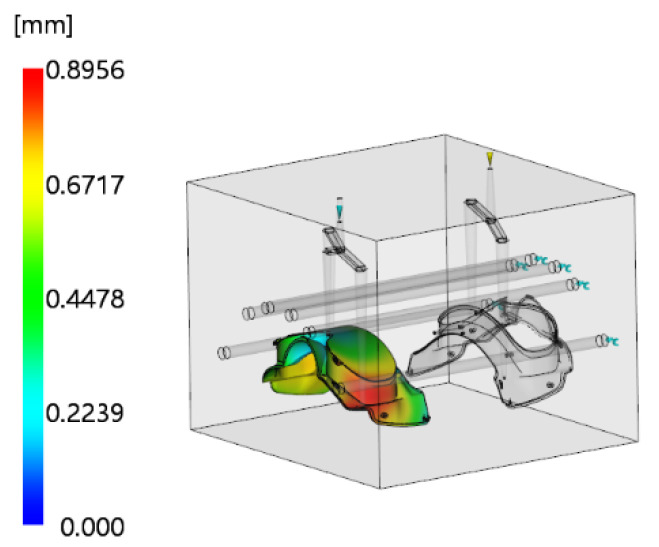
Warpage deformation contour plot after optimization.

The optimal parameters were substituted into Moldflow software for simulation, yielding a warpage deformation of 0.8956 mm, as shown in Figure 19. The relative error between this value and the predicted value (0.875 mm) is only 2.4%, verifying the accuracy of the BP-SSA optimization model. Compared with the initial simulation result of 2.792 mm, the warpage deformation is reduced by 67.9%, indicating effective control of the defect.

**Figure 20:**
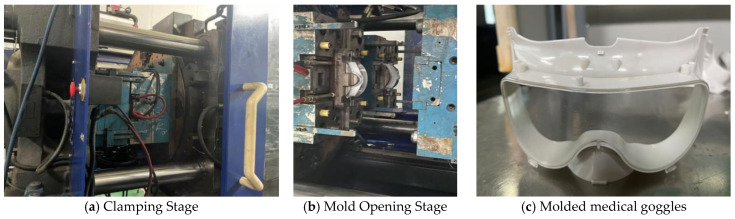
Mold trial verification.

Mold trial verification was conducted based on the parameters obtained from global optimization, as shown in Figure 20. Five production samples were randomly selected, and their warpage deformation was measured using a SEREIN Croma Classic Series coordinate measuring machine (CMM, Model: 8106). The average value was calculated and compared with the simulated value from Moldflow software, with the measurement results presented in Table 7. The average warpage deformation of the five samples is 0.944 mm, and the error relative to the optimized simulation result is only 5.4%. The main cause of the error is the slight uneven shrinkage of the samples during cooling to room temperature after demolding. The mold trial results demonstrate that the optimized process parameters possess excellent engineering practicality and can stably produce high-quality goggles.

## 4. Conclusions

With the aim of solving the problem of medical goggles needing to meet multiple performance requirements (e.g., splash resistance, leakage prevention, lightweight comfort, durability, and non-toxicity), while considering that traditional processes such as single-color injection molding and assembly are difficult to adapt, this study focuses on the optimization of the two-color injection molding process for medical goggles. Through material selection, CAE simulation modeling, intelligent optimization of process parameters, and mold trial verification, a complete high-quality molding solution has been developed. The main research conclusions are as follows:

(1) A suitable two-color injection molding material combination was determined: Based on the performance requirements of medical goggles and the principle of material compatibility, the first-shot frame material was selected as Apec 1745 PC (with high impact resistance and chemical corrosion resistance, suitable for frequent disinfection scenarios), and the second-shot lens material was Makrolon 2858 PC. Moldflow overmolding simulation verification showed that there were no unfused areas at the interface of this material combination, and their PVT properties and viscosity characteristics were similar. This effectively avoids defects such as debonding and delamination, reduces the risk of warpage deformation, and meets the core requirements of medical goggles for structural stability and light transmittance.
(2) A high-precision two-color injection molding CAE simulation model was established: Moldflow 2019 software was used to build a 3D simulation model. Through dual-domain mesh repair and 3D mesh generation, 841,420 tetrahedral elements (minimum layer count of 6) were obtained, and key mesh quality indicators (maximum aspect ratio of 2.97, maximum volume ratio of 9.41, etc.) all met the simulation requirements. The injection sequence of “frame as the first shot and lens as the second shot” was determined to avoid thermal damage to the transparent lens caused by high-temperature melt. The gating system (double gates for the frame and a single side gate for the lens) and cooling system (8 mm diameter cooling channels with a spacing of 20 mm and a distance of 16 mm from the cavity wall) were optimally designed to ensure uniform melt filling and reasonable distribution of temperature and stress fields, providing a reliable simulation basis for process optimization.
(3) An efficient process parameter optimization model was established: Taking the mold and melt temperatures, holding pressures, and holding times of the two shots as the seven design variables, and warpage deformation as the optimization objective, an *L*_32_ (7^4^) orthogonal test was designed to obtain 32 sets of process parameter-warpage deformation data. A BP neural network model was constructed to describe the nonlinear relationship between parameters and quality, and the SSA was used to optimize the network weights and thresholds, forming a BP-SSA intelligent optimization model. The MAPE of this model is only 2.28%, which is significantly better than that of the single BP neural network (14.36). It successfully avoids the local optimum problem and improves the prediction accuracy.
(4) The engineering practicality of the optimized process was verified: The optimal process parameters obtained by the BP-SSA model are as follows: mold temperature of 130 °C, first-shot melt temperature of 311 °C, second-shot melt temperature of 310 °C, first-shot holding pressure of 83 MPa, second-shot holding pressure of 70 MPa, first-shot holding time of 14 s, and second-shot holding time of 8 s. Simulation verification shows that the warpage deformation of the goggles under these parameters is 0.8956 mm, which is 67.9% lower than the initial simulation result (2.792 mm). Mold trials were conducted using a Haitian IA-2500 two-color injection molding machine. The average warpage deformation of five randomly selected samples is 0.944 mm, with a relative error of only 5.4% compared with the simulation result. The error originates from slight uneven shrinkage during the cooling stage after demolding, indicating that the optimized process can stably produce high-quality medical goggles.

The integrated method of “material selection-CAE simulation-orthogonal test-BP-SSA intelligent optimization” proposed in this study effectively solves the warpage defect problem in the two-color injection molding of medical goggles. It provides technical support for the large-scale and high-standard manufacturing of thin-walled, transparent, and multi-material medical products, and can be extended to the field of multi-material injection molding products with more complex structures.

## Figures and Tables

**Table 1:** Mesh quality.

Item	Requirement	Actual Value	Item	Requirement	Actual Value
Mesh Type	Tetrahedron	Tetrahedron	Maximum Aspect Ratio	<50	25.4
Connected Regions	2	2	Folded Faces	0	0
Minimum Refinement Layers	6	6	Inverted Tetrahedral Angles	0	0
Mesh Quantity (×10^4^)	84.333		Maximum Draft Angle	<180°	169.4°
	First Shot: 51.933		Maximum Edge Length Ratio	<3	2.97
	Second Shot: 32.409		Maximum Volume Ratio	<20	9.41

**Table 2:** Factors and levels.

Molding Parameters	A	B	C	D	E	F	G
Minimum Value	100	310	290	68	68	14	7
Maximum Value	130	340	320	97	97	17	10

**Table 3:** Neural network model parameter settings.

Item	Parameters
Topology Structure	Input layer, hidden layer, output layer
Number of Nodes per Layer	Input layer: 7, hidden layer: 8, output layer: 1
Activation Function	Hidden layer: tansig, output layer: purelin
Number of Training Epochs	1000
Learning Rate	0.01
Minimum Training Target Error	0.0001
Display Frequency	Display once every 25 training epochs
Training Method	Conjugate Gradient Algorithm

**Table 4:** Parameters of the two-color injection molding machine.

Item	Barrel A	Barrel B
Screw Diameter/mm	40	32
Nozzle Diameter/mm	3	2.5
Injection Pressure/MPa	225	225
Injection Rate/g·s^−1^	111	75
Theoretical Injection Volume/cm^3^·s^−1^	251	122

**Table 5:** Orthogonal test data table.

Test No.	A	B	C	D	E	F	G	K
1	100	310	290	68	68	14	7	2.858
2	100	310	300	78	97	17	9	2.917
3	100	320	310	97	68	15	9	3.19
4	100	320	320	88	97	16	7	3.32
5	100	330	290	88	78	17	8	3.46
6	100	330	300	97	88	14	10	3.286
7	100	340	310	78	78	16	10	3.271
8	100	340	320	68	88	15	8	3.232
9	110	310	290	97	88	17	8	2.847
10	110	310	300	88	78	14	10	2.83
11	110	320	310	67	88	16	10	3.012
12	110	320	320	88	78	15	8	2.382
13	110	330	290	78	97	14	7	3.224
14	110	330	300	68	68	17	9	1.958
15	110	340	310	88	97	15	9	2.831
16	110	340	320	97	68	16	7	3.336
17	120	310	290	68	97	15	10	2.125
18	120	310	300	78	68	16	8	1.429
19	120	320	310	97	97	14	8	1.895
20	120	320	320	88	68	17	10	1.597
21	120	330	290	88	88	16	9	2.061
22	120	330	300	97	78	15	7	2.382
23	120	340	310	78	88	17	7	2.697
24	120	340	320	68	78	14	9	2.105
25	130	310	290	97	78	16	9	2.228
26	130	310	300	88	88	15	7	1.934
27	130	320	310	68	78	17	7	0.981
28	130	320	320	78	88	14	9	1.429
29	130	330	290	78	68	15	10	0.963
30	130	330	300	68	97	16	8	1.853
31	130	340	310	88	68	14	8	2.086
32	130	340	320	97	97	17	10	1.717

**Table 6:** Comparison between actual values and predicted values.

Test No.	Actual Value/mm	BP Predicted Value/mm	BP-SSA Predicted Value/mm	BP Absolute Error/%	BP-SSA Absolute Error/%
1	2.83	3.105	2.697	9.72	4.70
2	2.382	2.207	2.375	7.35	0.29
3	1.429	1.87	1.456	30.86	1.89
4	3.336	2.897	3.235	13.16	3.03
5	1.895	2.098	1.923	10.71	1.48
Average Error	-	-	-	14.36	2.28

**Table 7:** Comparison between mold trial product results and optimized simulation results.

Actual Warpage Deformation/mm						Optimized Warpage Deformation/mm	Relative Error/%
Sample 1	Sample 2	Sample 3	Sample 4	Sample 5	Average Value		
0.95	0.93	0.91	1.02	0.91	0.944	0.8956	5.4

## Data Availability

The original contributions presented in this study are included in the article. Further inquiries can be directed to the corresponding author.
